# Influence of routine exercise on the peripheral immune system to prevent and alleviate pain

**DOI:** 10.1016/j.ynpai.2023.100126

**Published:** 2023-03-21

**Authors:** Joseph B. Lesnak, Giovanni Berardi, Kathleen A. Sluka

**Affiliations:** a Department of Neuroscience and Center for Advanced Pain Studies, University of Texas at Dallas, Richardson, TX, USA; b Department of Physical Therapy & Rehabilitation Sciences, University of Iowa, Iowa City, IA, USA

**Keywords:** Exercise, Immune system, Pain, Analgesia, Cytokines, Macrophage

## Abstract

Routine physical activity reduces the onset of pain and exercise is a first line treatment for individuals who develop chronic pain. In both preclinical and clinical research regular exercise (routine exercise sessions) produces pain relief through multiple mechanisms such as alterations in the central and peripheral nervous system. More recently, it has been appreciated that exercise can also alter the peripheral immune system to prevent or reduce pain. In animal models, exercise can alter the immune system at the site of injury or pain model induction, in the dorsal root ganglia, and systemically throughout the body to produce analgesia. Most notably exercise shows the ability to dampen the presence of pro-inflammatory immune cells and cytokines at these locations. Exercise decreases M1 macrophages and the cytokines IL-6, IL-1β, and TFNα, while increasing M2 macrophages and the cytokines IL-10, IL-4, and IL-1ra. In clinical research, a single bout of exercise produces an acute inflammatory response, however repeated training can lead to an anti-inflammatory immune profile leading to symptom relief. Despite the clinical and immune benefits of routine exercise, the direct effect of exercise on immune function in clinical pain populations remains unexplored. This review will discuss in more detail the preclinical and clinical research which demonstrates the numerous ways through which multiple types of exercise alter the peripheral immune system. This review closes with the clinical implications of these findings along with suggestions for future research directions.

## Highlights

• In rodents routine exercise prevents and reduces pain by altering the immune system.
• In rodents regular exercise decreases pro-inflammatory cytokines and immune cells.
• In rodents routine exercise increases anti-inflammatory cytokines and immune cells.
• In humans regular exercise may promote a systemic anti-inflammatory immune profile.

## 1 Introduction

Physical inactivity increases all-cause mortality and is a risk factor for the development of obesity, cardiovascular disease, diabetes, cancer, dementia, and depression (Biswas et al., 2015, Fischer et al., 2007, Lahart et al., 2015, Nocon et al., 2008, Paffenbarger et al., 1994, Rovio et al., 2005, Tuomilehto et al., 2001, Wolin et al., 2009). Physical inactivity is also a risk factor for the development of chronic pain conditions (Landmark et al., 2011, Landmark et al., 2013). Repeated exercise as an intervention is commonly prescribed for conditions such as low back pain, osteoarthritis, and fibromyalgia with moderate to strong evidence of effectiveness due to favorable effects in reducing pain severity and improving physical function, cognition, sleep, mental health and more (Bidonde et al., 2017, Geneen et al., 2017, Qaseem et al., 2017, Brosseau et al., 2017, Brosseau et al., 2017, Busch et al., 2011, Busch et al., 2013). To understand how routine exercise prevents and alleviates pain; research has been carried out to identify mechanisms of exercise induced analgesia. This research has demonstrated that exercise has several systemic effects that reduce pain at the level of the brain, spinal cord, and in the periphery and have been reviewed elsewhere (Lesnak and Sluka, 2020, Lima et al., 2017, Sluka et al., 2018).

There has been a recent surge in research surrounding the role of the peripheral immune system in the development and maintenance of chronic pain (Laumet et al., 2019, Totsch and Sorge, 2017, Tan et al., 2021, Baral et al., 2019). In preclinical research, genetic strains that limit immune system function and immune cell depletion techniques have implicated a role of the immune system in pain development. Athymic or *Rag1-/-* mice lack functional T cells and have reduced pain hypersensitivity following induction of neuropathic pain (Laumet et al., 2019). Similarly, depletion of macrophages from the gastrocnemius muscle prior to induction of pain prevents the onset of muscle hyperalgesia and depletion of neutrophils attenuates the development of paw hyperalgesia following paw incision (Carreira et al., 2013, de Azambuja et al., 2021, Gong et al., 2016, Gregory et al., 2016). In individuals with chronic pain, there is often increases in circulating pro-inflammatory cytokines and involvement of the immune system in disease etiology has been implicated in fibromyalgia, osteoarthritis, rheumatoid arthritis, peripheral neuropathy, low back pain, and complex regional pain syndrome (Alivernini et al., 2020, Held et al., 2019, Uçeyler et al., 2007, Ernberg et al., 2018, Teodorczyk-Injeyan et al., 2018, Bäckryd et al., 2017, Hysing et al., 2019, Totsch and Sorge, 2017, Uçeyler et al., 2007, Merriwether et al., 2021). Upstream mediators suggest ATP, lactate, and hydrogen ions activate receptors (P2X4, P2X7, ASIC3) on macrophages to induce release of inflammatory cytokines and enhance pain (Gregory et al., 2015, Oliveira-Fusaro et al., 2020). Therefore, researchers have explored if repeated exercise modulates the peripheral immune system to produce analgesia. Once again, repeated exercise produces systemic changes throughout the body by altering the immune systems response to pain in both preclinical and clinical research. The purpose of this review is to summarize the findings on routine exercises role in modulating the peripheral immune system to prevent or alleviate pain.

## 2 Animal studies

Animal models are commonly used to study mechanisms of exercise induced analgesia. These models frequently use aerobic forms of regular exercise such as treadmill training, swimming, or voluntary wheel running (Lesnak and Sluka, 2020), however recently a resistance training model was developed which was effective at preventing muscle pain (Lesnak et al., 2022). The following papers discussed in this review, all found that routine exercise was able to prevent or reduce hyperalgesia in various pain models including inflammatory, muscle, neuropathic, and osteoarthritic. In summary, regular exercise modulates immune response to pain at the multiple sites, including at site of injury, in the dorsal root ganglia (DRG), and systemically throughout the body (Table 1).

**Table 1:** Summary of animal literature investigating changes in the peripheral immune system in response to pain model induction following either pre-treatment or post-treatment exercise. CFA = Complete Freund’s Adjuvant, OA = Osteoarthritis, MIA = Monosodium Iodoacetate, CCI = Chronic Constriction Injury, I/R = Ischemia and Reperfusion, ACL = Anterior Cruciate Ligament, M = Male, F = Female, VWR = Voluntary Wheel Running, DRG = Dorsal Root Ganglia, PBMC = Peripheral Blood Mononuclear Cells.

**Impact of Exercise on the Peripheral Immune Response in Rodent Pain Models**					
Reference	Pain Model	Animals	Exercise Model	Location of Assessment	Immune Impact
**Site of Injury**					
*Swelling*					
(Pitcher et al., 2017)	Hindpaw CFA	M Long Evans Rats	Post Treatment VWR	Hindpaw	↔ Swelling
(Ludtke et al., 2020)	Hindpaw CFA	M Swiss Mice	Post Treatment Swimming	Hindpaw	↓ Swelling
(Shi et al., 2018)	Tibial Fracture	M C57BL/6 Mice	Post Treatment VWR	Hindpaw	↔ Swelling
(Jablonski et al., 2020)	Gout	M + F Balb/c Mice	Pretreatment Treadmill	Ankle	↓ Swelling; *no sex difference analysis
*Progression of Physiological Changes*					
(Allen et al., 2017)	Knee OA - MIA	M Sprague-Dawley Rats	Post Treatment Treadmill	Tibia	↓ Trabecular bone loss
(Castrogiovanni et al., 2019)	Knee OA - ACL transection	M Wistar Rats	Post Treatment Treadmill	Femoral Articular Cartilage	↓ Cartilage degeneration
(Lesnak et al., 2022)	Muscle - Activity Induced	M + F C57BL/6 Mice	Pretreatment VWR	Gastrocnemius Muscle	↓ Transcriptional immune response; *females only
(Cobianchi et al., 2010)	Neuropathic - Sciatic CCI	M CD1 Mice	Pre and Post Treatment Treadmill	Sciatic Nerve	↑ GAP43 and Cdc2 in sciatic nerve
*Immune Cell Number and Phenotype*					
(de Azambuja et al., 2021)	Muscle - Carrageenan	M Swiss Mice	Pretreatment Swimming	Gastrocnemius Muscle	↓ Number of macrophages, ↓ M1 macrophages, ↑ M2 macrophages
(Lesnak et al., 2022)	Muscle - Activity Induced	M + F C57BL/6 Mice	Pretreatment VWR	Gastrocnemius Muscle	↔ Number of macrophages, ↓ M1 macrophages, ↑ M2 macrophages, ↓ CD3 T cells; *both sexes
(Honda et al., 2022)	Muscle - Joint Immobilization	M Wistar Rats	Post Treatment Electrically Stimulated Muscle Contractions	Gastrocnemius Muscle	↓ Number of macrophages
(Grace et al., 2016)	Neuropathic - Sciatic CCI	M Sprague-Dawley Rats	Pretreatment VWR	Sciatic Nerve	↓ Number of macrophages, ↓ M1 macrophages, ↑ M2 macrophages
(Bobinski et al., 2018)	Nueropathic - Sciatic Crush	M Swiss and Balb/c Mice	Post Treatment Treadmill	Sciatic Nerve	↔ Number of macrophages, ↓ M1 macrophages, ↑ M2 macrophages
(Jablonski et al., 2020)	Gout	M + F Balb/c Mice	Pretreatment Treadmill	Sciatic Nerve	↓ Number of macrophages, ↓ Number of neutrophils; *no sex difference analysis
(Appleyard et al., 2021)	Endometriosis	F Sprague Dawley Rats	Pre and Post treatment VWR	Vesicles and Mesenteric Fat	↓ Number of macrophages, ↓ Number of mast cells
(Fuentes et al., 2021)	Stress Induced Perigenital Pain	M C57BL/6 Mice	Post treatment VWR	Bladder and Prostate	↓ Number of mast cells
(Ishikawa et al., 2019)	Knee OA - Kaolin and Carrageenan	M Wistar Rats	Post Treatment Electrically Stimulated Muscle Contractions	Knee Joint Synovium	↓ Number of macrophages
Cytokines					
(Ross et al., 2018)	Muscle - I/R	M Swiss Mice	Pretreatment VWR	Forepaw Muscles	↓ IL-1β
(de Azambuja et al., 2021)	Muscle - Carrageenan	M Swiss Mice	Pretreatment Swimming	Gastrocnemius Muscle	↓ IL-1β, ↑ IL-10
(Bobinski et al., 2011)	Nueropathic - Sciatic Crush	M Swiss Mice	Pretreatment Treadmill	Sciatic Nerve	↓ IL-1β, TNFα; ↔ IL-6R
(Chen et al., 2015)	Neuropathic - Diabetic	M Wistar Rats	Post Treatment Treadmill	Sciatic Nerve	↓ TNFα, IL-6, ↑ IL-10
(Chen et al., 2012)	Neuropathic - Sciatic CCI	M Sprague-Dawley Rats	Post Treatment Treadmill or Swimming	Sciatic Nerve	↓ IL-1β, TNFα
(Huang et al., 2017)	Neuropathic - Sciatic CCI	M Sprague-Dawley Rats	Post Treatment Treadmill	Sciatic Nerve	↓ TNFα, IL-6, ↑ IL-10
(Tsai et al., 2017)	Neuropathic - Sciatic CCI	M Sprague-Dawley Rats	Post Treatment Treadmill	Sciatic Nerve	↓ TNFα, IL-6, ↑ IL-10
(Bobinski et al., 2018)	Nueropathic - Sciatic Crush	M Swiss Mice	Post Treatment Treadmill	Sciatic Nerve	↑ IL-4, IL-1ra
(Jablonski et al., 2020)	Gout	M + F Balb/c Mice	Pretreatment Treadmill	Ankle Synovial Tissue	↓ IL-1β; *no sex difference analysis
(Castrogiovanni et al., 2019)	Knee OA - ACL transection	M Wistar Rats	Post Treatment Treadmill	Knee Joint Synovium	↓ IL-1β, TNFα, ↑ IL-10, IL-4; ↔ IL-6
(Shi et al., 2018)	Tibial Fracture	M C57BL/6 Mice	Post Treatment VWR	Hindpaw Skin	↓ IL-1β, CCL2
Dorsal Root Ganglia					
(Grace et al., 2016)	Neuropathic - Sciatic CCI	M Sprague-Dawley Rats	Pretreatment VWR	L4-5 DRG	↓ Number of macrophages, ↓ ATF3
(Chhaya et al., 2019)	Neuropathic - C5 contusion	F Sprague Dawley Rats	Post Treatment Treadmill	C7-8 DRG	↓ Number of macrophages
(Almeida et al., 2015)	Neuropathic - Sciatic Ligation	M Balb/c Mice	Post Treatment Swimming	L4-5 DRG	↓ NGF, BDNF
(Lopez-Alvarez et al., 2015)	Neuropathic - Sciatic Transection	F Sprague Dawley Rats	Post Treatment Treadmill	L3 DRG	↓ NGF; ↓ NKCC1
(Chen et al., 2014)	Incisional	M Sprague-Dawley Rats	Post Treatment Treadmill	L3-5 DRG	↓ Substance P; ↓ IL-1β, IL-6
(Yoon et al., 2015)	Neuropathic - Diabetic	M Sprague-Dawley Rats	Post Treatment Treadmill	L4-6 DRG	↓ TRPV1, TRPM8 channels; ↓ IL-1β, TNFα
(Ma et al., 2019)	Neuropathic - Diabetic	Male Sprague-Dawley Rats	Post Treatment Treadmill	L4-6 DRG	↓ IL-1R, IL-6R, TNFR1; ↓ IL-1β, IL-6, TNFα
(Mifflin et al., 2019)	EAE	M + F C57BL/6 Mice	Pretreatment VWR	L4-6 DRG	↓ Calcium response to KCl; *females only
Systemic Changes					
(Grace et al., 2016)	Neuropathic - Sciatic CCI	M Sprague-Dawley Rats	Pretreatment VWR	Blood and PBMCs	↓ CCL2, CCL3, CXCL1; ↑ IL-10; ↓ Release of IL-1β and IL-10 from LPS stimulated PBMCs
(Mifflin et al., 2019)	EAE	M + F C57BL/6 Mice	Pretreatment VWR	Spleenocytes	↓ Release of TNFα from MOG 33–55 stimulated spleenocytes; *females only
(Appleyard et al., 2021)	Endometriosis	F Sprague Dawley Rats	Pre and Post Treatment VWR	Blood	↓ CXCL1, CCL5, CXCL5
(Jablonski et al., 2020)	Gout	M + F Balb/c Mice	Pretreatment Treadmill	Blood	↓ TLR2 on neutrophils, CXCL1; *no sex difference analysis
(Cooper et al., 2017)	High-Fat Diet	M C57BL/6 Mice	VWR during high-fat diet	Blood	↓ IL-1β and IL-6 mRNA

### 2.1 Site of injury or pain model induction

**Fig. 1:**
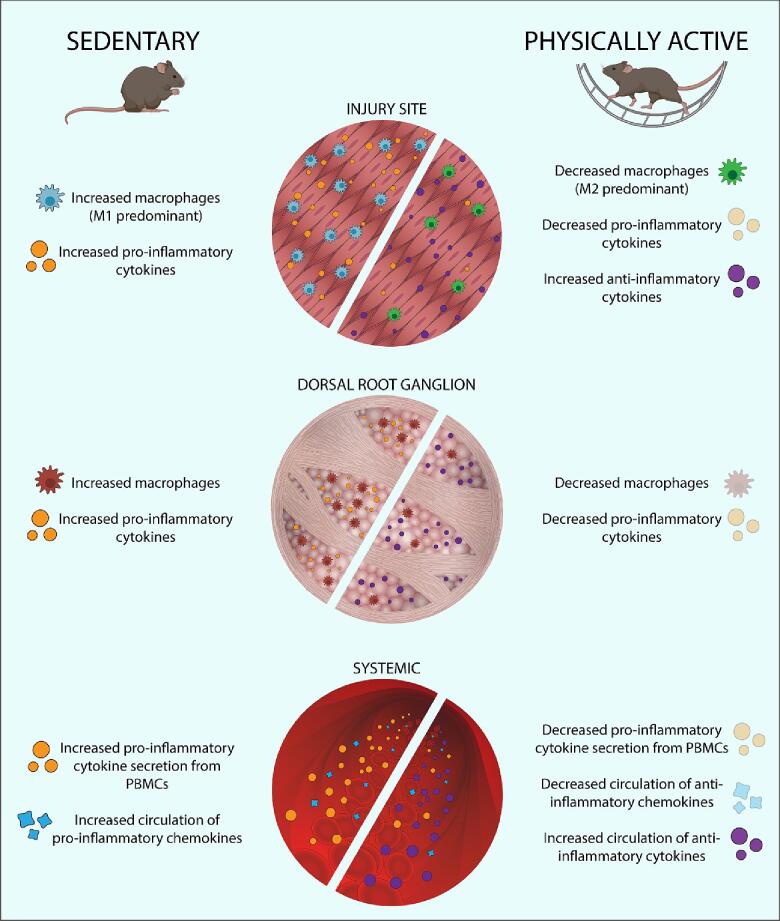
Summary of the impact of regular exercise on the peripheral immune system to prevent and alleviate pain. Exercise works at the site of injury, dorsal root ganglia, and systemically to decrease pro-inflammatory macrophages and cytokines and increase anti-inflammatory macrophages and cytokines.

The most extensive research on exercises ability to modulate the peripheral immune system has been done at the site of injury or pain model induction. This work demonstrates routine exercise can impact swelling, physiological changes initiated by pain model, number and phenotype of local immune cells, and presence of local cytokines in response to pain model induction (Fig. 1).

#### 2.1.1 Swelling

Exercise shows mixed results when it comes to its ability to prevent or alleviate swelling after induction of pain. Injection of Complete Freund’s adjuvant (CFA) into the hindpaw of rodents produces a robust immune response resulting in swelling of the limb and mechanical hypersensitivity. Voluntary wheel running performed after injection of CFA causes no significant reduction in swelling in rats (Pitcher et al., 2017); however, in mice, swimming initiated after injection of CFA results in lower levels of edema when compared with sedentary animals (Ludtke et al., 2020). Interestingly rats with CFA hindpaw injections given access to voluntary wheel running, ran comparable distances as rats who received CFA vehicle injections suggesting the lack of reduction in edema was not caused by a lack of running wheel activity induced by CFA. Voluntary wheel running initiated after induction of a tibial fracture pain model does not reduce temperature or edema of the injured hindpaw (Shi et al., 2018). Lastly, treadmill training for two weeks prior to induction of a gout model, reduces the amount of ankle swelling in mice compared with sedentary animals (Jablonski et al., 2020). The conflicting results regarding the impact of exercise on swelling could be due to the pain model studied, species utilized, and exercise parameters such as type and volume. Despite the lack of reduction in edema in the cited studies, exercise was still shown to reduce hindpaw hypersensitivity and weightbearing asymmetry in response to CFA (Pitcher et al., 2017) and tibial fracture (Shi et al., 2018) suggesting analgesic effects of exercise are not solely produced through a reduction in swelling.

#### 2.1.2 Progression of physiological changes initiated by pain models

Routine exercise slows or prevents disease progression in various animal pain models. Models of knee osteoarthritis are induced either through intraarticular injection of an insult such as monosodium iodoacetate (MIA), 3% kaolin and carrageenan or by anterior cruciate ligament (ACL) transection. These OA models cause progressive joint pathology demonstrated through reductions in cartilage thickness, structural alterations in the synovium, and trabecular bone loss. Treadmill training initiated 10 days after MIA injection reduces the amount of trabecular bone loss in rats (Allen et al., 2015). While treadmill training initiated 2 weeks after ACL transection slows cartilage degeneration in rats (Castrogiovanni et al., 2019).

Muscle pain models applied at the gastrocnemius muscle are induced by intramuscular injections of insults such as acidic saline or carrageenan, through fatiguing muscle contractions, or by long term ankle immobilization. Induction of muscle pain causes a robust immune response at the muscle level demonstrated through both transcriptional alterations and increases in immune cells (Gong et al., 2016, Gregory et al., 2015, Lesnak et al., 2022). RNA sequencing performed on the gastrocnemius muscle 24 hours after induction of activity-induced muscle pain demonstrates upregulation of several immune system pathways including chemokine signaling, viral protein interaction, toll-like receptor signaling, and NOD-like receptor signaling in both sedentary male and female mice. This work also reveals a female specific activation of the antigen processing and presentation pathway in sedentary females only. Eight weeks of voluntary wheel running prior to induction of muscle pain causes a dampening of the transcriptional immune system response in females but not males, with a complete prevention in the transcriptional activation of the antigen processing and presentation pathway (Lesnak et al., 2022). This suggests repeated exercise performed prior to muscle pain can blunt the immune system response to later insults to prevent pain.

Lastly, chronic constriction injury (CCI) or a crush of the sciatic nerve is routinely used as a model of neuropathic pain in rodents and results in significant damage to the sciatic nerve and subsequent hindpaw mechanical hypersensitivity. Treadmill training performed 2 weeks prior and 3 to 7 days post CCI causes an accelerated regeneration of the injured sciatic nerve demonstrated through higher amounts of growth associated protein 43 and Cdc2 in the sciatic nerves of mice (Cobianchi et al., 2010). Similarly, treadmill training initiated after sciatic nerve crush results in a higher number of myelinated fibers which also demonstrated increased fiber diameter and myelin sheath thickness in mice (Bobinski et al., 2011).

#### 2.1.3 Immune cell numbers and phenotype

Routine exercise alters the amount and phenotype of immune cells at the site of induction of the pain model. Induction of muscle pain produces an increase in the number of macrophages in the gastrocnemius muscle (de Azambuja et al., 2021, Gong et al., 2016, Gregory et al., 2015, Honda et al., 2022, Lesnak et al., 2022). Swimming prior to carrageenan injection attenuates the number of macrophages in the gastrocnemius muscle of mice (de Azambuja et al., 2021); however, voluntary wheel running prior to activity-induced muscle pain has no impact on the number of macrophages in the gastrocnemius (Lesnak et al., 2022). Voluntary wheel running prevents an increase in CD3 T cells in the gastrocnemius muscle in response to activity-induced muscle pain in male and female mice (Lesnak et al., 2022). Lastly, electrically stimulated muscle contractions initiated after joint immobilization decreases the number of macrophages in the gastrocnemius muscle of rats when compared with sedentary animals (Honda et al., 2022).

Macrophages are plastic and can have a pro-inflammatory phenotype (M1) or an anti-inflammatory phenotype (M2) (Stout et al., 2005). M1 macrophages are responsible for phagocytic activity and secrete pro-inflammatory cytokines such as IL-1β, IL-6, and TNFα, while M2 macrophages are responsible for tissue repair and secrete anti-inflammatory cytokines such as IL-10, IL-4 and IL-1ra (Stout et al., 2005, Mosser and Edwards, 2008, Murray et al., 2014, Murray and Wynn, 2011). In pain free mice, a single bout of treadmill training or 8 weeks of voluntary wheel running increases the number of M2 macrophages in the gastrocnemius (Ikeda et al., 2013, Leung et al., 2016). In sedentary animals, induction of muscle pain through either carrageenan injection or fatiguing muscle contractions combined with acidic saline increases M1 macrophages (de Azambuja et al., 2021, Lesnak et al., 2022). However, animals with prior swimming or voluntary wheel running see a predominant increase in M2 macrophages in the gastrocnemius muscle after induction of the pain model (de Azambuja et al., 2021, Lesnak et al., 2022). Again, this suggests that prior exercise can impact how the immune system responds to later insults to prevent the onset of muscle pain.

Similar results are seen in models of neuropathic pain induced via sciatic nerve injuries. In sedentary animals, there are increases in the number of macrophages in the sciatic nerve with a higher predominance of the M1 phenotype following nerve injury (Bobinski et al., 2018, Grace et al., 2016). Voluntary wheel running performed prior to sciatic nerve injury decreases the total number of macrophages and increases the proportion of M2 macrophages in the sciatic nerve (Grace et al., 2016). Treadmill training initiated 3 days after sciatic nerve injury causes a shift in macrophage phenotype in the sciatic nerve at the site of injury toward a higher prevalence of M2 macrophages but did not reduce total number of macrophages (Bobinski et al., 2018). This shift in macrophage phenotype is prevented in IL-4 knockout mice suggesting exercises ability to promote M2 macrophages is mediated through release of IL-4 (Bobinski et al., 2018).

Routine exercise alters local immune cell quantities in less studied pain models. Treadmill training performed prior to induction of a gout model decreases the number of macrophages and neutrophils into the ankle joint synovium of mice (Jablonski et al., 2020). In a rat model of endometriosis, voluntary wheel running two weeks prior and after model induction results in decreased numbers of mast cells and macrophages in vesicles and mesenteric fat (Appleyard et al., 2021). Voluntary wheel running attenuates the number of mast cells in the bladder and prostate in mice with perigenital hypersensitivity produced by neonatal maternal separation (Fuentes et al., 2021). Lastly, in a knee OA model induced by intraarticular injection of 3% kaolin and carrageenan, quadriceps muscle stimulation initiated after induction of the pain model reduced the number of macrophages in the knee joint synovium in rats compared with sedentary animals (Ishikawa et al., 2019). Thus, these data consistently shows regular exercise modulates the immune system in uninjured animals, and after induction of pain in a variety of animal models.

#### 2.1.4 Alterations in cytokines

Regular exercise can also alter the balance of pro and anti-inflammatory cytokines at the site of injury. Most commonly studied is the ability of exercise to reduce pro-inflammatory cytokines IL-1β, TNFα, and IL-6 and increase the levels of anti-inflammatory cytokines IL-4, IL-10, and IL-1ra. In a mouse model of muscle pain produced by limb ischemia and reperfusion, prior voluntary wheel running prevents increases in IL-1β in forepaw muscles (Ross et al., 2010). Following carrageenan injection into the gastrocnemius muscle there are increases in pro-inflammatory cytokine IL-1β in the muscle which is prevented by prior swimming exercise (de Azambuja et al., 2021), and swimming exercise increases the anti-inflammatory cytokine IL-10 in the muscle (de Azambuja et al., 2021). IL-10 seems to be a crucial mediator in exercises ability to prevent muscle pain as the analgesic effects of voluntary wheel running prior to muscle pain induction is prevented by IL-10 antibody treatment and local intramuscular delivery of IL-10 attenuates the onset of muscle pain (Leung et al., 2016). Similarly, a single eccentric exercise bout increases IL-10 in the gastrocnemius muscle and knockdown of IL-10 receptors in the DRG blocks exercises ability to prevent muscle pain to a subsequent overuse exercise session (Alvarez et al., 2017).

Similarly, in models of neuropathic pain induced by sciatic nerve injury or diabetic neuropathy, sedentary animals show an increase in pro-inflammatory cytokines IL-1β, TNFα, and IL-6, and a decrease in anti-inflammatory cytokines following induction of pain in the injured nerve (Bobinski et al., 2011, Chen et al., 2012, Chen et al., 2014, Huang et al., 2017, Tsai et al., 2017). When treadmill training is performed prior to sciatic nerve injury, there are decreases in pro-inflammatory cytokines IL-1β and TNFα in the injured nerves (Bobinski et al., 2011). When initiated after induction of neuropathic pain, treadmill training and swimming both reduce IL-1β, TNFα, and IL-6 in the injured nerves (Chen et al., 2012, Chen et al., 2014, Huang et al., 2017, Tsai et al., 2017) and increases the anti-inflammatory cytokines IL-10, IL-4, and IL-1ra in the injured nerve (Bobinski et al., 2018, Chen et al., 2012, Huang et al., 2017, Tsai et al., 2017). Blockade of IL-4 via IL-4 knockout mice or treatment with an IL-4 antibody, prevents the analgesic effects of treadmill training (Bobinski et al., 2018). This suggests increases in anti-inflammatory cytokines, particularly IL-4, within the injured nerve appear to mediate the ability of regular exercise to alleviate neuropathic pain.

In an animal model of gout, prior treadmill training attenuates the increase in IL-1β in the ankle joint synovium of mice (Jablonski et al., 2020). In knee OA produced by ACL transection, treadmill training initiated after injury reduces levels of IL-1β and TNFα and increases levels of IL-4 and IL-10 in the knee joint synovium of rats (Castrogiovanni et al., 2019). In a mouse model of intervertebral disc degeneration, voluntary wheel running results in decreased levels of IL-1β and TNFα in the multifidus muscle (James et al., 2018). Lastly, voluntary wheel running initiated after a tibial joint fracture reduces levels of IL-1β and CCL2 in hindpaw skin of mice (Shi et al., 2018).

In sum routine exercise demonstrates the ability to alter local immune response to pain when performed before or after the insult. Exercise modulates the local response by reducing swelling, slowing disease progression, decreasing numbers of immune cells, and by shifting the local environment to an anti-inflammatory setting through manipulation of macrophage phenotype and presence of cytokines.

### 2.2 Dorsal root ganglia

Repeated exercise also modulates the immune system at the level of DRG through altering the presence of immune cells, attenuating the number of neurotrophic and transcription factors, normalizing functional activity, and reducing pro-inflammatory cytokines (Fig. 1). The DRG serves as the cell body for nociceptors in the periphery which carry sensory information to the spinal cord, and thus serves as an integral location for studying pain mechanisms. First, exercise attenuates the number of macrophages in the DRG in animal models of neuropathic pain. Voluntary wheel running prior to CCI of the sciatic nerve reduces macrophage accumulation in the DRG of rats (Grace et al., 2016). Similarly, a model of neuropathic pain induced by a unilateral C5 contusion results in increased macrophages in the DRG of rats and treadmill training performed afterwards, reduces the number of macrophages (Chhaya et al., 2019).

Next, regular exercise reduces the presence of neurotrophic and transcription factors in the DRG. Voluntary wheel running prior to CCI of the sciatic nerve attenuates increases in the transcription factor ATF3 in rats (Grace et al., 2016). Treadmill training and swimming initiated after sciatic nerve ligation reduces DRG levels of neurotrophic factors NGF and BDNF in response to sciatic nerve ligation in mice and rats (Almeida et al., 2015, Lopez-Alvarez et al., 2015). Finally, treadmill training reduces levels of substance P in the DRG following induction of post-incisional pain in rats (Chen et al., 2015).

Routine exercise modulates expression levels of ion channels and receptors on DRGs. In rats with diabetic induced neuropathic pain, treadmill training normalizes levels of TRPV1 and TRMP8 cation channels (Yoon et al., 2015), and decreases expression of pro-inflammatory cytokine receptors IL-1R, IL-6R, and TNFR1 in the DRG (Ma et al., 2019). Also, treadmill training after sciatic nerve ligation prevents the increase in chloride transporter NKCC1 in the DRG of rats (Lopez-Alvarez et al., 2015). Finally, cultured DRGs from sedentary male and female mice with experimental autoimmune encephalomyelitis (EAE) demonstrate increased amplitude of calcium response to KCl stimulation (Mifflin et al., 2019). In animals with EAE with access to voluntary running wheels, the calcium response to KCl stimulation is decreased but in females only (Mifflin et al., 2019).

Lastly, repeated exercise modulates levels of pro-inflammatory cytokines in the DRG. Treadmill training reduces DRG levels of IL-1β, IL-6, and TFNα in rats with diabetic induced neuropathic pain (Ma et al., 2019, Yoon et al., 2015). Also, treadmill training reduces DRG levels of IL-1β and IL-6 following induction of post-incisional pain in rats (Chen et al., 2015). Thus, exercise modulates immune function at the level of the DRG through alterations in presence of immune cells, transcription, neurotrophic factors, excitatory neurotransmitters, ion channels and pro-inflammatory cytokines.

### 2.3 Systemic changes

Induction of pain models can produce systemic alterations in immune system function including circulating chemokines and cytokines which is modulated by routine exercise (Fig. 1**)**. Following sciatic CCI in rats, cultured PBMCs from blood stimulated with LPS produce higher amounts of IL-1β and IL-10 when compared with pain free animals (Grace et al., 2016). Voluntary wheel running prior to CCI injury prevents the increased release of IL-1β and IL-10 from LPS stimulated PBMCs (Grace et al., 2016). In a mouse model of EAE, cultured immune cells from the spleen increased secretion of the pro-inflammatory cytokines of IFNγ, TNFα, and IL-17a when stimulated with myelin oligodendrocyte glycoprotein (MOG_35-55_); voluntary wheel running prevents the MOG_35-55_ stimulated increase of TNFα, in females only (Mifflin et al., 2019).

Sciatic nerve CCI results in increased circulating levels of the chemokines CCL2, CCL3, and CXCL1 in rats (Grace et al., 2016). Voluntary wheel running prior to CCI, attenuates circulating levels of these chemokines and elevates levels of the anti-inflammatory cytokine IL-10 (Grace et al., 2016). Similarly, prior voluntary wheel running, prevents the systemic elevation of the chemokines CXCL1, CCL5, and CXCL5 in response to induction of endometriosis in rats (Appleyard et al., 2021). In mice, treadmill training prior to induction of gout, decreases the expression of TLR2 on neutrophils and levels of CXLC1 in circulation (Jablonski et al., 2020). Lastly, voluntary wheel running reduces circulating levels of IL-1β and IL-6 mRNA in mice with high-fat diet induced pain (Cooper et al., 2017). Thus, repeated exercise shows its ability to produce systemic immune effects suggesting its beneficial effects are not limited to the group of muscles subject to training.

## 3 Human studies

In healthy human subjects, acute bouts of exercise transiently increase inflammation, while regular exercise may act as an anti-inflammatory treatment (Walsh et al., 2011, Campbell and Turner, 2018, Klasson et al., 2022, Sellami et al., 2021, Zhao et al., 2012, Chastin et al., 2021, Shimizu et al., 2008, Simpson et al., 2020, Ferreira et al., 2018, Cantó et al., 2018, Scheffer and Latini, 2020, Nieman and Pence, 2020, Nieman and Wentz, 2019, Suzuki, 2018, Cerqueira et al., 2019). Acute bouts of exercise elicit a cascade of inflammatory events measured by changes in PBMC counts, lymphocyte subpopulations (T cells, B cells, natural killer (NK) cells), granulocyte, neutrophil and NK cell activity, lymphocyte proliferation, and cytokine levels in plasma (Nieman, 1997, Gabriel and Kindermann, 1997, Pedersen, 1991, Gonçalves et al., 2019, Moldoveanu et al., 2001). Some suggest acute bouts of exercise may transiently redistribute immune cells to peripheral tissues, leading to immune system adaptations and improved immunocompetence (Campbell and Turner, 2018, Simpson et al., 2015, Simpson et al., 2020). On the contrary, repeated bouts of physical exercise may enhance the immune response, reinforce antioxidative capacity, reduce oxidative stress, and increase the efficiency of energy generation, thus inducing an anti-inflammatory state and reducing the incidence of inflammatory diseases (Laddu et al., 2021, Scheffer and Latini, 2020, Pedersen and Hoffman-Goetz, 2000). Multiple factors may influence the inflammatory response following exercise, including exercise type, intensity, duration, and clinical conditions including individuals with chronic pain (Nieman et al., 2012, Moldoveanu et al., 2001, Allen et al., 2015, Bigley and Simpson, 2015, Silveira et al., 2016, Hennigar et al., 2017, Peake et al., 2008). For example, single bouts of prolonged, high-intensity exercise impair T cell, NK cell, and neutrophil function, alter cytokine balance, and blunt immune responses to primary and recall antigens in vivo in healthy individuals. In contrast, single and routine bouts of low-to-moderate intensity exercise enhance immune function including reduced number of senescent T cells, increased T cell proliferation, lower levels of circulating inflammatory cytokines, and increased neutrophil phagocytic activity (Simpson et al., 2015, Spielmann et al., 2011, Simpson et al., 2012, Shinkai et al., 1995, Pedersen and Bruunsgaard, 2003, Yan et al., 2001). Therefore, the evidence in healthy humans suggests low-to-moderate intensity exercise may lead to beneficial effects on immune function which in theory should lead to improved pain and treatment outcomes. The beneficial effect of routine low-to-moderate intensity exercise on immune function in healthy humans perhaps mimics the reported animal literature in prior sections which primarily consist of voluntary wheel running or graded increases in exercise exposure. However, the clinical literature is comprised of exercise protocols ranging from low-to-high intensity exercise which are summarized below and in Table 2.

**Table 2:** Summary of human literature investigating changes in immune function with acute and chronic exercise. RA = Rheumatoid Arthritis, HC = Healthy Control, PBMC = Peripheral Blood Mononuclear Cell, ESR = Erythrocyte Sedimentation Rate, CRP = C-Reactive Protein, HIIT = High-Intensity Interval Training, VO2 peak = Peak Oxygen Consumption, NK = Natural Killer, DAS28 = Disease Activity Score 28, CDAI = Clinical Disease Activity Index, HAQ-DI = Health Assessment Questionnaire - Disability Index, PGIC = Patient Global impression of Change, OA = Osteoarthritis, HA = Hyaluronan, COMP = Cartilage Oligomeric Matrix Protein, CPII = C-Propeptide of Type II Procollagen, CTX-II = C-Telopeptide of Type II Collagen, C2C = Type II Collagen Cleavage Neoepitope, LPS = Lipopolysaccharide, VAS = Visual Analogue Scale, MFI = Multidimensional Fatigue Inventory, PPT = Pressure Pain Threshold, FIQ = Fibromyalgia Impact Questionnaire, BDNF = Brain Derived Neurotrophic Tactor, NGF = Nerve Growth Factor, FM = Fibromyalgia; For additional references not in text see (Andersson et al., 2020, Bartlett et al., 2018, Bjersing et al., 2012, Bote et al., 2013, Bote et al., 2014, Cheng et al., 2015, Chua et al., 2008, Helmark et al., 2010, Hunt et al., 2013, Jablochkova et al., 2019, Oliveira et al., 2017, Ortega et al., 2012, Ortega et al., 2009, Salm et al., 2019, Samut et al., 2015, Simão et al., 2012, Wåhlén et al., 2022).

Condition	Ref	Participants	Exercise Mode	Tissue	Findings
RA and aging	(Rall et al., 1996)	8 RA, 8 age-matched HC, 8 young HC	2x/week, 12 weeks Progressive resistance strength training	Whole blood	↔, no change in PBMC subsets, IL-1B, TNF-a, IL-6, IL-2, lymphocyte proliferation, DTH response
RA, prediabetes	(Andonian et al., 2018)	12 people with RA 9 people with prediabetes	Alternating low (50–60% heart rate reserve) and high (80–90% heart rate reserve) intensity treadmill intervals, 3x/week for 10 weeks	Plasma, muscle biopsy	↔ plasma galectin-3, skeletal muscle cytokines,
RA	(Bartlett et al., 2018)	12 inactive adults with RA	10 week high-intensity interval walking, 3x/week, 30 min sessions	Whole blood	↓ ESR ↔ CRP, IL-1β, IL-6, CXCL-8, TNF-α, IL-10 ↑ neutrophil migration and bactericidal function (adaptive immunity) HIIT improved the balance of inflammatory pathologically related CD16-expressing monocytes to CD16 negative monocytes Significant correlation observed between changes in relative VO2peak and IL-10
RA	(Baslund et al., 1993)	18 individuals with RA	Randomly allocated to 1) 8-week progressive bicycle program 2) control group	Whole blood	↔ PBMCs, proliferative response, NK cell activity, IL-1a, IL-1B, IL-6
RA	(Andersson et al., 2020)	49 individuals with RA	Randomly allocated to 20-weeks of 1)Exercise group = resistance + aerobic exercise performed at moderate- to high intensity, 3x/week (n = 24) 2) active control group (n = 25)	Whole blood	↔ CRP, DAS28, CDAI, HAQ-DI, PGIC ↓ Tregs ↑ IL-2 (supernatent) ↔ total CD4 Tcells, IFNy, IL-17a, IL-10, total white blood cells, neutrophils, lymphocytes, proportion of CD3 Tcells, CD8 Tcells ↓ Bregs ↔ total CD19 + population
Migraine	(Oliveira et al., 2017)	20 women with episodic migraine	Randomly assigned to 1) 12-week aerobic exercise program, 30 min, 3 days/week, (walking on a treadmill) or 2) inactive control	Plasma	↓ IL-12p70 ↔ TNF-α, IL-1β, IL-6, IL-8, IL-10
Low back pain	(Cheng et al., 2015)	30 individuals with low back pain	5 min of stretching + 10 min of strengthening of the back musculature, 3x/week, 4 weeks	Whole blood, plasma	↓ TLR-4 mRNA expression ↓ IFN-γ and IP-10 concentrations ↑ SIRT1, FoxO1 and FoxO3 expression ↓ p53, PPAR-γ and PGC-1α expression ↓ NF-κB activity ↓ IL-1B, IL-6, IL-8, TNF-a ↑ ROS
OA, obese	(Nicklas et al., 2005)	Overweight/obese older adults with knee OA 275 participants completed the first 6 months 252 participants completed the entire 18-month intervention 218 provided blood samples	316 people assigned to 1) exercise, 2) dietary weight loss, 3) exercise + dietary weight loss, 4) control Exercise = 3x/week, 18 months Consisted of an aerobic phase (15 min), a resistance-training phase (15 min), a second aerobic phase (15 min), and a cool-down phase (15 min)	Whole blood	↔, no effects on IL-6, TNF-α, or the TNFR1 and TNFR2 polymorphisms on responses to exercise for any of the outcomes Physical function was related to genetic variation in three different cytokine genes (IL-6, TNFα, and TNFR2)
OA, obese	(Chua et al., 2008)	Overweight/obese adults with knee OA	316 people assigned to 1) exercise (n = 46), 2) dietary weight loss (n = 48), 3) exercise + dietary weight loss (n = 46), 4) control (n = 53) Exercise = 3x/week, 18 months Consisted of an aerobic phase (15 min), a resistance-training phase (15 min), a second aerobic phase (15 min), and a cool-down phase (15 min)	Whole blood	↔ serum levels of HA, COMP, and TGF-β1 remained relatively stable during the 18-month intervention (exercise) period
OA	(Helmark et al., 2010)	29 females with knee OA	Single session of LE resistance exercise	Peri-synovial and intra-articular fluid	↑ IL-10, IL-6, IL-8, TNF-α
OA	(Simão et al., 2012)	32 elderly with knee OA	Randomly allocated to 1) squat ex on vibratory platform, 2) squat ex no platform (3x/week for 12 weeks)	Plasma	↓ sTNFR1 in the vibration group ↔ sTNFR2
OA	(Hunt et al., 2013)	17 individuals with radiographically-confirmed medial tibiofemoral OA	10-week physiotherapist led exercise, 4 days/week Strengthening targeting the hip abductors, quadriceps, hamstrings.	Serum, Urine	↓ serum COMP ↔ HA, COMP, CPII ↔ urine CTX-II, C2C
OA	(Samut et al., 2015)	42 individuals, >50 years of age with a diagnosis of knee OA	Randomly allocated to 1) isokinetic exercise program (3 days/week, 6 weeks), 2) aerobic ex (3 days/week, 6 weeks), 3) control group	Serum	↔ TNF-a, IL-6, CRP (both groups)
Fibromyalgia	(Ortega et al., 2009)	14 females with fibromyalgia, 13 matched-controls	3 weekly 60-minute sessions, 4 months Aquatic fitness program consisting of aerobic, stretching, and strengthening	Whole blood	↓ IL-8, IFN-γ (pro-inflammatory cytokines), CRP, cortisol ↔ TNF-α, IL-1β, IL-2, IL-6, IL-4, IL-10
Fibromyalgia	(Ortega et al., 2012)	9 females with fibromyalgia, 9 age-matched controls	2 – 60-minute sessions per week, 8 months Aquatic fitness program included stretching, aerobic, strengthening	Whole blood	↓ spontaneous and LPS-induced production of IL-1β at 8 months ↓ spontaneous TNF-α at 4 months ↓ spontaneous and LPS-induced TNF-α at 8 months, ↑ spontaneous and LPS-induced IL-6 at 4 months ↓ spontaneous and LPS-induced IL-6 at 8 months ↓ spontaneous IL-10 at 4 and 8 months ↑ LPS-induced IL-10 at 4 and 8 months ↔ CRP at 4 months ↓ CRP at 8 months
Myalgia	(Mackey et al., 2011)	42 women with clinically diagnosed trapezius myalgia	Specific strength training group, n = 18, performed high-intensity strength training with five dumbbell exercises specifically for the shoulder and neck muscles, 20 min sessions, 3x/week Cycle ergometer group, n = 16, 20 min sessions, 3x/week Control group, n = 8, health information only	Muscle biopsy	↑ macrophages and satellite cells with strengthening group
Fibromyalgia	(Bote et al., 2013)	8 women with rheumatologist diagnosed fibromyalgia	Single bout of moderate intensity cycling	Whole blood, serum, plasma	↑ IL-8, cortisol, noradrenaline, and eHsp72 (heat shock protein) in healthy women ↓ IL-8, cortisol, noradrenaline (without the differences being significant), eHsp72 in FM ↑ release of all the cytokines (IL-1β, TNF-α, IL-6, IL-10, and IL-18) by monocytes from healthy women ↓ release of inflammatory cytokines by monocytes from FM patients (although without significant differences for IL-10); ↔ TNF-a
Fibromyalgia	(Bote et al., 2014)	10 women with rheumatologist diagnosed fibromyalgia	Aquatic exercise program, 8 months, 2 sessions/week, 60 min/session	Plasma, serum	↔ serum IL-8, plasma noradrenaline, neutrophils at 4 months ↓ neutrophil chemotaxis, IL-8, noradrenaline at 8 months
Fibromyalgia	(Ernberg et al., 2016)	24 females with fibromyalgia	15 weeks of progressive resistance exercise twice per week	Interstitial muscle via microdialysis of the vastus lateralis Plasma	↔ plasma or dialysate IL-1β, TNF, IL-6, and IL-8
Fibromyalgia	(Ernberg et al., 2018)	125 females with fibromyalgia allocated to treatments 130 female matched controls	Treatment groups: 1) 15-weeks progressive resistance exercise, 2x/week focused on strengthening and flexibility focused on the LE, core stability; 2) Control = relaxation therapy	Plasma	FM at baseline: ↑ IL1-B, IL-2, IL-6, TNF-a, IP-10, eotaxin ↑ IL-1ra with intervention (exercise & relaxation) ↑ IL-1β in the relaxation group Weak correlations existed between the changes in most cytokines and changes in clinical variables such as VAS, MFI, PPT, and FIQ
Fibromyalgia	(Jablochkova et al., 2019)	75 women with fibromyalgia 25 healthy controls for baseline measures only	Treatment groups: 1) 15-weeks progressive resistance exercise, 2x/week focused on strengthening and flexibility focused on the LE, core stability; 2) Control = relaxation therapy	Plasma	Baseline: ↑ BDNF, ↓ NGF ↔ BDNF, NGF with resistance or relaxation
Fibromyalgia	(Salm et al., 2019)	28 women with fibromyalgia	Performed aquatic exercise program (6 weeks, 3x/week, 18–50 min sessions) consisting of stretching, aerobic, strengthening. • Group 1 = aquatic Ex + Far-Infrared therapy (n = 14) • Group 2 = aquatic ex + placebo (n = 14)	Serum	↓ IL-6 in both groups ↔ TNF, IL-10 in both groups
Fibromyalgia	(Wåhlén et al., 2022)	Fibromyalgia = 40 females (23 resistance ex, 17 relaxation) HC = 25 (resistance ex only)	Randomized to 15 weeks resistance training or relaxation therapy	Plasma	• 484 proteins analyzed • 28 proteins involved in regulation of immune system process, muscle structure development, and response to stress were able to discriminate between FM and CON
Fibromyalgia	(Bjersing et al., 2012)	49 women with fibromyalgia	Randomized to 15 weeks (2x/week, 40–45 min per session) of 1) mod-to-high intensity Nordic Walking (n = 26) or 2) low-intensity walking (n = 23)	Serum, CSF	↔IGF-1 at 15 weeks, but ↓ at 30-wk follow-up in both groups ↔IGFBP-3 in both groups Higher level of IGF-1 at baseline indicated less pain during exercise in fibromyalgia
Fibromyalgia	(Ross et al., 2010)	165 individuals with fibromyalgia	Acute exercise task = Modified Balke Treadmill Exercise Protocol (exhaustive exercise)	Serum	Those with an abnormal growth hormone response to exhaustive exercise demonstrated: ↑ IL-1a, IL-6, IL-8 ↔ IL-1β, IL-1RA, IL-10 and TNF-α

Multiple chronic pain conditions are linked to etiology associated with changes in immune system function including fibromyalgia, osteoarthritis, rheumatoid arthritis, peripheral neuropathy, low back pain, and complex regional pain syndrome (Alivernini et al., 2020, Bäckryd et al., 2017, Ernberg et al., 2016, Held et al., 2019, Hysing et al., 2019, Teodorczyk-Injeyan et al., 2018, Uçeyler et al., 2007). While the effects of exercise on immune function have been investigated and described in healthy populations, the evidence in chronic pain populations is less clear due to conflicting evidence, varying exercise protocols, and limited study.

### 3.1 Acute/single bouts of exercise

Despite the relevance to exercise prescription there has been limited investigation into the impact of acute exercise on immune function and pain in clinical populations. Prior study suggests individuals with fibromyalgia who have a proinflammatory state of elevated cytokines IL-1α, IL-6, and IL-8 at baseline experience a defective stress response to high-intensity exercise as measured by reduced circulating growth hormone and increased pain following exercise, suggesting the inflammatory status of patients influences exercise response (Ross et al., 2018). However, the change in circulating cytokines following exercise and the relation to clinical symptoms are unclear. The impact of acute bouts of low-to-moderate intensity exercise on immune function are equivocal. Bote et al. (2013) showed a decrease in IL-8 concentration in circulating serum and stimulated release of IL-1β, TNF-α, and IL-6 by monocytes following a single bout of moderate intensity cycling in fibromyalgia; however, the changes in clinical pain were not evaluated. In individuals with knee osteoarthritis, an acute bout of moderate-intensity resistance exercise increases the anti-inflammatory cytokine IL-10 locally within the synovium and peri-articular area of the knee joint (Helmark et al., 2010); again, clinical pain was not assessed. In contrast, local effects of exercise are not observed in fibromyalgia, as a 20-minute bout of low-intensity exercise did not change pro-inflammatory cytokines (IL-1β, IL-6, IL-8, and TNF) in the exercising muscle (Christidis et al., 2015). A systematic review of 6 articles examining a single bout of exercise showed unclear results for a directional preference circulating cytokines in fibromyalgia (Andrade et al., 2018). Of the available evidence, the relation between changes in circulating cytokines to changes in pain in clinical populations following a single bout of exercise is limited. Further investigation into the immune response following single bouts of exercise in chronic pain is needed to examine the positive and negative effects on the immune system and how these relate to symptoms.

### 3.2 Routine exercise

Since regular exercise has the capacity to modulate immune function to enhance anti-inflammatory activity of immune cells in healthy people and animal models (Walsh et al., 2011, Calcaterra et al., 2022, Klasson et al., 2022, Campbell and Turner, 2018, Zhao et al., 2012, Shimizu et al., 2008, Sellami et al., 2021, Chastin et al., 2021, Oliveira-Fusaro et al., 2020, Sluka and Gregory, 2015, Gregory et al., 2018, Gregory et al., 2015, Leung et al., 2016, Leung et al., 2016, da Silva et al., 2015, Nijs et al., 2014, Polli et al., 2019, Clark et al., 2017, Liu and Wang, 1987, Kohut et al., 2001, Baek et al., 2020, Cannon and Kluger, 1984), it is important to determine if regular exercise modulates the immune system in chronic pain populations. Prior randomized controlled trials using various modes of exercise ranging from aerobic (cardiorespiratory), muscle strengthening, stretching, and aquatic based exercise show varying effects on immune function. Ortega et al (2009) showed 8 months of aquatic exercise reduced monocyte evoked release of proinflammatory markers (IL-1, IL-6, TNFα), circulating C-reactive protein, and increased anti-inflammatory marker (IL-10) in individuals with fibromyalgia. Thus, four months of routine exercise improved the balance of pro- and anti-inflammatory cytokine production and lead to better regulation of monocyte function in individuals with fibromyalgia. There was also improvement in quality of life and function, however direct correlations to immune changes were not investigated. Furthermore, Bote et al (2014) showed 8-months of routine aquatic exercise reduced neutrophil chemotaxis, circulating IL-8 and noradrenaline at 8 months, while there were no changes at 4 months in individuals with fibromyalgia, suggesting longer duration of routine exercise may be necessary to induce changes in immune function that promote anti-inflammatory adaptations. Concomitant improvements in quality of life support the hypothesis that exercise-induced changes in inflammatory function may impact clinical symptoms and function. Despite these promising results, others have found routine exercise did not change various markers of immune function (Table 2) including PBMC subsets in RA, (Baslund et al., 1993, Rall et al., 1996) cytokine gene polymorphisms in knee OA, (Nicklas et al., 2005) and plasma cytokines in migraine (Oliveira-Fusaro et al., 2020), muscle in RA (Andonian et al., 2018) and muscle in fibromyalgia (Ernberg et al., 2018). Variation in the prior literature can be attributed to multiple factors including focusing systemic vs local effects, small sample sizes, and different pain conditions. On the other hand, in women with trapezius myalgia, there is an increased numbers of macrophages in muscle which could be related to the regenerative myogenic response to strength training (Mackey et al., 2011).

In addition to factors such as exercise type, intensity, duration, and tissue type which may impact outcomes, the mixed results from the available literature may be attributable to low sample size in the majority of studies, use of mixed populations of immune cells, use of varying methods to investigate immune function, and use of different stimuli to evoke cytokine release from immune cells. In addition, the potential influence of biological sex on changes in immune function following exercise in humans is unknown as most studies have included females only or a limited number of males in their sample. Despite these limitations, preliminary studies show that exercise can alter systemic cytokines and reduce systemic inflammation, a proposed mechanism of chronic pain in humans. Additionally, there has been a lack of investigation into the relation of changes in clinical pain measures to immune system changes.

## 4 Clinical implications & future direction

### 4.1 Animal research

The animal research demonstrates that regular exercise can modulate the immune system response to induction of pain at the site of injury, in the DRG, and in the systemic circulation. This suggests that repeated exercise bouts produce whole body effects, and its benefits are not restricted to the location of training. Also, several types of exercise programs are beneficial at preventing or reducing pain suggesting patient preference should be heavily considered when prescribing exercise programs to help increase adherence. Lastly, routine exercise shows positive benefits when performed either before or after onset of pain. Therefore, exercise programs should still be followed in the absent of pain to help prevent pain exacerbations. While this review highlighted several articles showing regular exercise-induced changes in the immune response, many questions remain regarding the underlying mechanisms through which exercise alters immune function. Future research should focus on upstream and downstream mechanisms for how exercise modulates immune phenotype and alters cytokine release. Understanding these mechanisms could identify novel therapeutic targets and assist with exercise prescription for the treatment of chronic pain. Also worth noting is the lack of research utilizing both sexes to study the effectiveness and mechanisms of exercise-induced analgesia. This is especially concerning in studies exploring the immune system since there are significant sex differences in immune mechanisms in the generation of pain (Baskozos et al., 2019, Cowie et al., 2019, Mecklenburg et al., 2022, Mogil, 2020, Plumb et al., 2023, Sorge et al., 2015). Of the papers that did include both males and females, sex differences were found in exercise immune mechanisms (Mifflin et al., 2019, Lesnak et al., 2022) and are highlighted above. Therefore, future work needs to include both male and female animals to further elucidate sex differences in exercises ability to modulate the immune system to prevent and alleviate pain.

### 4.2 Human research

Acute bouts of exercise and routine exercise training influence the immune response in individuals with chronic pain. The ability of the immune system to adapt towards an anti-inflammatory state with routine exercise intervention may provide promising outcomes for the treatment of chronic pain. In addition to pain, the anti-inflammatory effects of routine exercise may provide multiple health benefits including reduced cardiovascular disease, obesity, type 2 diabetes, risk of cancer, sarcopenia, and dementia. The available evidence does not support specific exercise guidelines that promote an anti-inflammatory state as multiple factors may influence the inflammatory response to acute and chronic exercise. Appraisal of the available literature suggests it may be particularly important to adjust the characteristics of and exercise programs in order to obtain an anti-inflammatory response and/or restore an optimal anti- and pro-inflammatory state. When managing patients with chronic pain, clinicians should consider initiating at low to moderate intensity exercise that is adapted to the individual patient to help lessen the pro-inflammatory response that is typically seen following acute bouts of high-intensity exercise. This is of particular importance when prescribing exercise to untrained individuals or those with limited physical capacity. An exercise program should be tailored to the individual needs and ability of the patient to help reduce barriers to routine physical activity and promote compliance to long-term intervention. The treatment goal should be to establish a long-term exercise program which improves pain, activities of daily living, quality of life, and immune function. In addition to exercise effects, a comprehensive treatment plan may lead to additive or synergistic effects on immune function with targeted pharmacological agents and dietary modification or supplementation. Future research should capitalize on advances in assessment of immune system and exercise immunology in relation to clinical and behavioral assessments in humans and animals. Further research investigating the link between the immune system and acute and chronic exercise in chronic pain conditions is needed to identify unique insights into potential mechanisms underlying the pathology of pain, therapeutic targets, and management strategies.

## 5 Conclusion

The available animal and human literature provide strong indication that routine exercise leads to adaptations in immune function which may relate to pain behaviors in animals and self-reported symptoms in humans. While single bouts of exercise may lead to transient increases in inflammation, routine exercise may be beneficial as an intervention or preventative treatment to promote an anti-inflammatory immune profile which can subsequently reduce pain. Thus, repeated bouts of exercise should be utilized as a first-line intervention for individuals with chronic pain due to its ability to positively impact various physiological systems to prevent and alleviate pain.

## Declaration of Competing Interest

The authors declare that they have no known competing financial interests or personal relationships that could have appeared to influence the work reported in this paper.
